# Heart dose and cardiac comorbidities influence death with a cardiac cause following hypofractionated radiotherapy for lung cancer

**DOI:** 10.3389/fonc.2022.1007577

**Published:** 2022-10-11

**Authors:** Kathryn Banfill, Azadeh Abravan, Marcel van Herk, Fei Sun, Kevin Franks, Alan McWilliam, Corinne Faivre-Finn

**Affiliations:** ^1^ Department of Clinical Oncology, The Christie National Health Service (NHS) Foundation Trust, Manchester, United Kingdom; ^2^ Division of Cancer Sciences, University of Manchester, Manchester, United Kingdom; ^3^ St James’s Institute of Oncology, Leeds Cancer Centre, Leeds, United Kingdom

**Keywords:** lung cancer, radiotherapy, cardiac toxicity, dose constraint, cardiac comorbidities

## Abstract

**Background:**

There is increasing evidence of cardiac toxicity of thoracic radiotherapy however, it is difficult to draw conclusions on cardiac dose constraints due to the heterogeneity of published studies. Moreover, few studies record data on cause of death. The aim of this paper is to investigate the relationship between conventional cardiac dosimetric parameters and death with cardiac causes using data from the UK national cause of death registry.

**Methods:**

Data on cancer diagnosis, treatment and cause of death following radical lung cancer radiotherapy were obtained from Public Health England for all patients treated at the Christie NHS Foundation Trust between 1/1/10 and 31/12/16. Individuals with metastatic disease and those who received multiple courses of thoracic radiotherapy where excluded. All patients who received > 45Gy in 20 fractions were included. Cardiac cause of death was defined as the following ICD-10 codes on death certificate: I20-I25; I30-I32; I34-I37; I40-I52. Cardiac V5Gy, V30Gy, V50Gy and mean heart dose (MHD) were extracted. Cumulative incidence of death with cardiac causes were plotted for each cardiac dosimetric parameter. Multi-variable Fine and Gray competing risk analysis was used to model predictors for cardiac death with non-cardiac death as a competing risk.

**Results:**

Cardiac dosimetric parameters were available for 967 individuals, 110 died with a cardiac cause (11.4%). Patients with a cardiac comorbidity had an increased risk of death with a cardiac cause compared with those without a cardiac comorbidity (2-year cumulative incidence 21.3% v 6.2%, p<0.001). In patients with a pre-existing cardiac comorbidity, heart V30Gy ≥ 15% was associated with higher cumulative incidence of death with a cardiac cause compared to patients with heart V30Gy <15% (2-year rate 25.8% v 17.3%, p=0.05). In patients without a cardiac comorbidity, after adjusting for tumour and cardiac risk factors, MHD (aHR 1.07, 1.01-1.13, p=0.021), heart V5Gy (aHR 1.01, 1-1.13, p=0.05) and heart V30Gy (aHR 1.04, 1-1.07, p=0.039) were associated with cardiac death.

**Conclusion:**

The effect of cardiac radiation dose on cardiac-related death following thoracic radiotherapy is different in patients with and without cardiac comorbidities. Therefore patients’ cardiovascular risk factors should be identified and managed alongside radiotherapy for lung cancer.

## Introduction

Lung cancer is the third most commonly diagnosed cancer in the UK and radiotherapy is used as the primary treatment for lung cancer in 20-55% of patients (1). One third of patients treated for lung cancer will have concomitant cardiovascular disease or have cardiovascular risk factors such as hypertension or diabetes (2–4).

Improvements in radiotherapy technology over the last 2 decades, including image guided radiotherapy (IGRT) and intensity modulated radiotherapy (IMRT), have led to more conformal radiotherapy, allowing the treatment of patients with larger tumours and better avoidance of organs at risk. The increase in radiotherapy conformality has facilitated the delivery of ablative doses for early-stage lung cancer showing high rates of local control and comparable outcomes to surgical resection in some patient groups (5, 6). The same success has not been achieved with increasing radiotherapy dose in patients with stage III lung cancer where the seminal RTOG 0617 trial of radiotherapy dose escalation failed to demonstrate a survival benefit compared to standard dose (7). The RTOG 0617 trial highlighted the heart as an organ at risk (OAR) in thoracic radiotherapy and reported that the volume of heart receiving ≥ 5Gy (V5Gy) or ≥30Gy (V30Gy) were associated with worse overall survival (7, 8). Following RTOG 0617, there have been many retrospective, single institution studies on cardiac toxicity in lung cancer (9, 10). The most common whole heart dose parameters examined and found to be significantly associated with overall survival are mean heart dose (MHD), heart V5Gy and heart V30Gy (9). The majority of the studies reported so far on cardiac toxicity have the end point of overall survival (8, 11), cardiac events (12–14), or non-cancer death (15). Most patients in these studies were treated with concurrent chemoradiotherapy in daily fractions of 1.8-2Gy.

It is clear that there is uncertainty in the cardiac dose parameters to be used in radiotherapy planning, the interaction with cardiac comorbidities and their association with subsequent cardiac events and survival. Furthermore, there is a paucity of data in patients treated with hypofractionated radiotherapy which is increasingly used worldwide for the treatment of patients with lung cancer. Therefore, the primary aim of our study is to examine if commonly used whole heart dose parameters (MHD, V5Gy, V30Gy and V50Gy) predict for death with a cardiac cause in a large cohort of patients with lung cancer treated with hypofractionated radiotherapy.

## Methods

### Databases

The National Cancer Registration and Analysis Service (NCRAS) is a longitudinal cancer registry that collects data on all people living in England who are diagnosed with cancer. Data on every primary tumour are collected from 162 National Health Service Providers and include data on: staging, pathology, systemic treatment, radiotherapy and hospital activity. NCRAS can be linked with cause of death data supplied by the Office for National Statistics (ONS) and Hospital Episode Statistics (HES) data on all admissions to NHS hospitals in England. Data are coded using the International Statistical Classification of Diseases and Related Health Problems 10th Revision (ICD-10) (10). In addition, data on deprivation is available from NCRAS using the Index of Multiple Deprivation (IMD) which is a measure of relative deprivation for small, fixed geographic areas of the UK. IMD classifies these areas into five quintiles based on relative disadvantage, with quintile 1 being the least deprived and quintile 5 being the most deprived.

Approval was granted to collect and analyse patient data for this study by the Leeds East Research Ethics Committee (18/YH/0058) and the UK Computer Aided Theragnostics (UKCAT) Research Database Management Committee (REC reference: 17/NW/0060). The UKCAT project is based at The Christie NHS Foundation Trust and automatically collects, pseudonymises and stores data from the trust’s electronic health record (16). Cohorts from both institutions were combined and linked with the national databases discussed above.

### Study population

All patients with stage I to IV lung cancer treated with radical radiotherapy at The Christie NHS Foundation Trust between 1/1/2010 and 31/12/2016 were identified using UKCAT. Patients were de-anonymised and the NHS numbers used to link with NCRAS data, and then pseudonymised again prior to analysis.

Data on patient sex, Eastern Co-operative Group performance status (PS), smoking status and radiotherapy were obtained from the UKCAT database. Tumour stage and histology, systemic treatment, (IMD quintile) and cause of death were obtained from NCRAS. Tumour stage was based on the 2010 Union for International Cancer Control TNM Classification of lung tumours version 7. Histology was based on the International Classification of Diseases for Oncology Version 3 (ICD-O3). If data on tumour stage or histology were missing from the NCRAS dataset, then the UKCAT dataset was used. If NCRAS coded the patient as having diagnosis based on imaging and no histology was recorded, this was considered a clinical diagnosis.

The HES dataset was used to identify patients who had a cardiac comorbidity recorded during a hospital admission from 1/1/2010 to 31/12/2016; defined using the following ICD-10 codes: I11; I13; I20-I25; I30-I52 (**Table 1**). A patient was defined as dying with a cardiac cause if the following ICD-10 codes were present in part 1 or part 2 of the medical certificate of cause of death (MCCD): I20-I25; I30-I32; I34-I37; I40-I52 (**Table 1**). Death certificates were available for patients who died prior to 30th November 2017, therefore follow-up was censored at this date.

**Table 1:** ICD10 codes to be used to identify cardiac comorbidities and death with a cardiac cause.

ICD10 Code	Meaning
	**Hypertensive Heart Disease**
I11	Hypertensive heart disease
I13	Hypertensive heart and renal disease
	**Ischaemic Heart Disease**
I20	Angina
I21	Acute myocardial infarction
I22	Subsequent myocardial infarction
I23	Complications after myocardial infarction
I24	Acute ischaemic heart disease
I25	Chronic ischaemic heart disease
	**Pericardial disease**
I30	Acute pericarditis
I31	Other diseases of pericardium
I32	Pericarditis in diseases classified elsewhere
	**Valve disease**
I33	Acute and subacute endocarditis
I34	Mitral valve disorder
I35	Aortic valve disorder
I36	Tricuspid valve disorder
I37	Pulmonary valve disorder
I38	Endocarditis, valve unspecified
I39	Endocarditis and heart valve disorder in diseases classified elsewhere
	**Myocardial disease**
I40	Acute myocarditis
I41	Myocarditis in diseases classified elsewhere
I42	Cardiomyopathy
I43	Cardiomyopathy in diseases classified elsewhere
	**Arrhythmia**
I44	Atrioventricular block and left bundle branch block
I45	Other conduction disorders
I46	Cardiac arrest
147	Paroxysmal tachycardia
I48	Atrial fibrillation
I49	Other cardiac arrhythmias
I50	Heart failure
I51	Complications and ill-defined descriptions of heart disease
I52	Other heart disorders in diseases classified elsewhere

Patients were included in the study if they were treated with hypofractionated radiotherapy to the lungs, defined as a dose >45Gy delivered in 20 fractions. Patients who received more than one course of thoracic radiotherapy and those with stage M1b disease were excluded.

### Radiotherapy planning and treatment

A 3-dimensional (3D) or 4-dimensional (4D) planning CT scan was carried out. In patients planned with 3D CT the gross tumour volume (GTV) was contoured and a 5mm margin was added for clinical target volume (CTV). The planning target volume was created by adding a 13mm sup/inf margin and 8mm margin radially to the CTV. In patients planned with a 4D CT, a motion adapted GTV was contoured using the maximum intensity projection and a 5mm margin added to create an internal target volume (ITV). A further 5mm isotropic margin was created from the ITV to create the planning target volume (PTV).

The heart was contoured to include the full extent of the pericardium from the superior aspect of the left pulmonary artery to the inferior aspect of the heart as described in the UK Stereotactic Ablative Radiotherapy (SABR) consortium guidance document (17). Cardiac contours were reviewed by one clinician (KB) and plans with cardiac contours that did not meet the guidelines were excluded from analysis.

Heart dose parameters of V30Gy < 40% and V40<30% were introduced during plan optimisation for this cohort in 2015. Other dose constraints used as part of the radiotherapy planning process include: spinal cord maximum dose < 44Gy, whole lungs-PTV V20Gy < 35% and lung-ITV (4D CT planning) or CTV (3D CT planning) mean dose < 20Gy. The following heart dose parameters were extracted from the radiotherapy planning data for patients with a validated heart contour: V5Gy, V30Gy, V50Gy and MHD.

### Statistical analysis

Key baseline characteristics were summarised based on history of cardiac comorbidities. Categorical variables were compared using a Chi-squared test and continuous variables using a Mann-Whitney Utest. We conducted Cox regression for all-cause mortality to assess overall survival differences for commonly used whole heart dose constraints: MHD, V5Gy, V30Gy and V50Gy. Plots of Schoenfeld residuals were performed to check for proportional hazards assumptions. We calculated cumulative incidence estimates of death with a cardiac cause with a cutpoint of 10Gy MHD based on previous publications (12–14). For cardiac V5Gy, V30Gy and V50Gy the median value was used as the cutpoint for calculating cumulative incidence estimates as previous literature has not defined a threshold value. The relationship between cardiac dose parameters and death with a cardiac cause were plotted using cumulative incidence estimates and compared using Fine and Gray competing risk regression with non-cardiac death as a competing risk. Multivariable Fine and Gray regression models were carried out separately for each cardiac dose parameter to avoid multi-collinearity. Variables were predefined for inclusion in the analysis based on known prognostic variables for survival in lung cancer (PS, stage, histology, tumour laterality and receipt of chemotherapy) and heart disease (sex, deprivation index and smoking status).Variables in which data on more than 25% of patients were missing were excluded from analysis Full death certificate information was not available for 27 patients and these were excluded from the Fine and Gray analysis.

## Results

### Clinical characteristics

**Figure 1:**
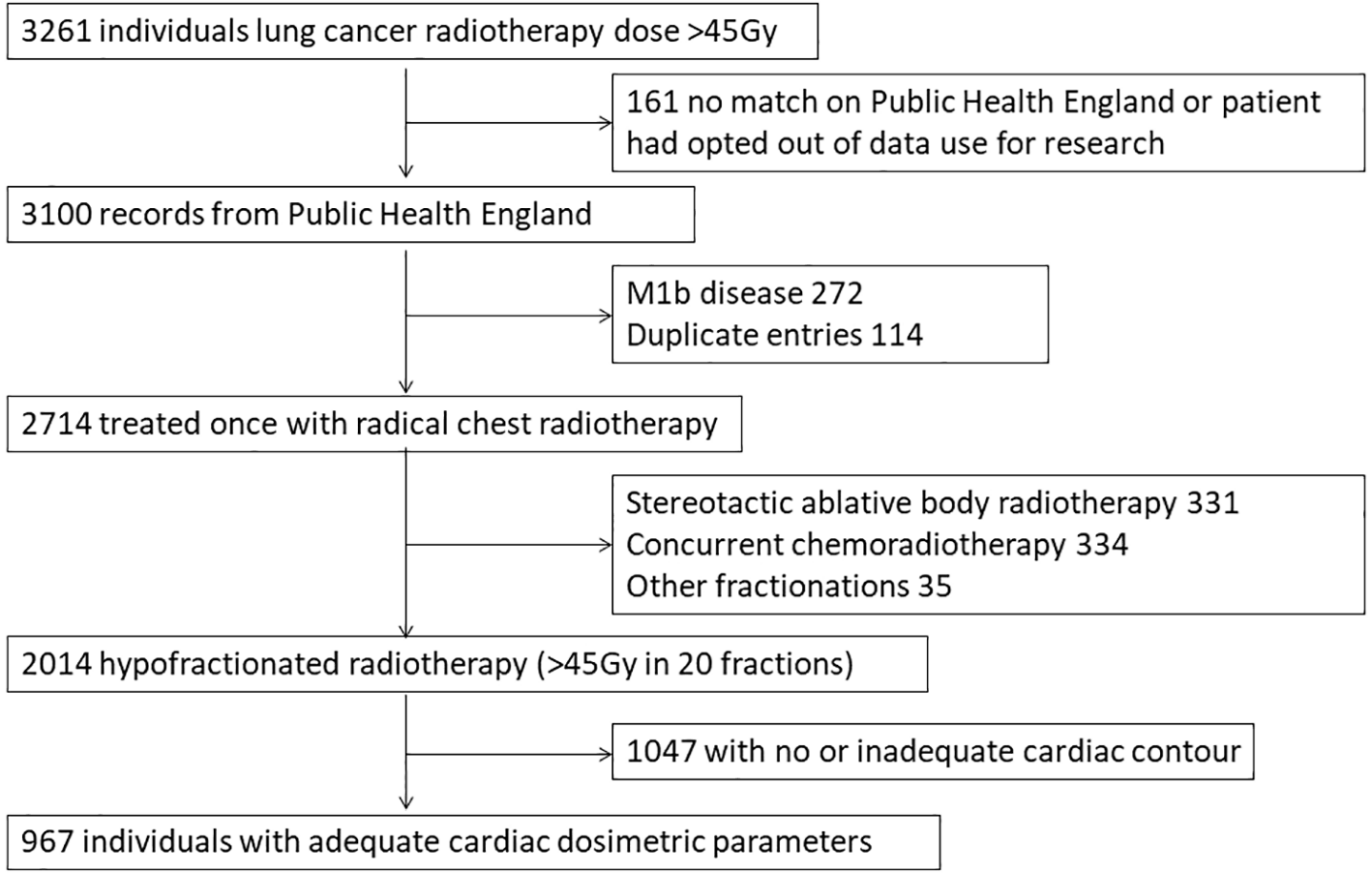
Flow diagram of patients included in final analysis.

Linked individual patient data were available from UKCAT and NCRAS for 3100 patients treated with radiotherapy for lung cancer between 2010 and 2016. Two thousand and fourteen patients were treated with hypofractionated radiotherapy and cardiac dosimetric parameters were available for 967 patients (**Figure 1**).

The clinical characteristics of the 967 patients treated with hypofractionated radiotherapy and had a available cardiac dosimetric parameters are shown in **Table 2**. Patients with at least one cardiac comorbidity were significantly older (median age 75.8 v 72.2 years). Significantly more patients with a cardiac comorbidity were male and were ex-smokers. The cohort of patients without a cardiac comorbidity contained more patients with N3 disease. There was no difference in the dosimetric parameters or the PTV volume between the 2 groups.

**Table 2:** Patient and treatment characteristics.

		No cardiac comorbidity n=675	Cardiac comorbidity n=292	Total	P
**Age at start of radiotherapy**	Median (IQR)	72.2 (13.3)	75.8 (10.7)	73.5 (12.6)	**<0.001**
**Sex**	Female	340 (50.4)	112 (38.4)	452 (46.7)	**0.001**
	Male	335 (49.6)	180 (61.6)	515 (53.3)	
**Performance Status**	0	55 (8.1)	20 (6.8)	75 (7.8)	0.348
	1	317 (47.0)	123 (42.1)	440 (45.5)	
	2	241 (35.7)	115 (39.4)	356 (36.8)	
	3	57 (8.4)	31 (10.6)	88 (9.1)	
	(Missing)	5 (0.7)	3 (1.0)	8 (0.8)	
**Histology**	NSCLC	481 (71.3)	223 (76.4)	704 (72.8)	0.256
	clinical diagnosis	93 (13.8)	34 (11.6)	127 (13.1)	
	SCLC	101 (15.0)	35 (12.0)	136 (14.1)	
**T stage**	T1	99 (14.7)	47 (16.1)	146 (15.1)	0.366
	T2	235 (34.8)	111 (38.0)	346 (35.8)	
	T3	175 (25.9)	80 (27.4)	255 (26.4)	
	T4	155 (23.0)	53 (18.2)	208 (21.5)	
	(Missing)	11 (1.6)	1 (0.3)	12 (1.2)	
**N stage**	N0	227 (33.6)	107 (36.6)	334 (34.5)	**0.022**
	N1	111 (16.4)	59 (20.2)	170 (17.6)	
	N2	223 (33.0)	99 (33.9)	322 (33.3)	
	N3	111 (16.4)	27 (9.2)	138 (14.3)	
	(Missing)	3 (0.4)	0 (0.0)	3 (0.3)	
**Deprivation**	least deprived	92 (13.6)	37 (12.7)	129 (13.3)	0.304
	2	70 (10.4)	28 (9.6)	98 (10.1)	
	3	96 (14.2)	53 (18.2)	149 (15.4)	
	4	149 (22.1)	74 (25.3)	223 (23.1)	
	most deprived	268 (39.7)	100 (34.2)	368 (38.1)	
**Smoking Status**	Current	196 (29.0)	54 (18.5)	250 (25.9)	**0.004**
	Ex-smoker	312 (46.2)	161 (55.1)	473 (48.9)	
	Never	10 (1.5)	7 (2.4)	17 (1.8)	
	Not known	157 (23.3)	70 (24.0)	227 (23.5)	
**Laterality**	Left	291 (43.1)	113 (38.7)	404 (41.8)	0.221
	Right	383 (56.7)	179 (61.3)	562 (58.1)	
	(Missing)	1 (0.1)	0 (0.0)	1 (0.1)	
**neo-adjuvant chemotherapy**	No	525 (77.8)	232 (79.5)	757 (78.3)	0.621
	Yes	150 (22.2)	60 (20.5)	210 (21.7)	
**MHD**	Median (IQR)	12.8 (1.8-24)	12.6 (1.2-24.1)	12.8 (1.7-23.9)	0.653
**Heart V5Gy**	Median	48.3	47.2	47.9	0.663
**Heart V30Gy**	Median	15.4	13.8	14.9	0.197
**Heart V50Gy**	Median	4.1	4.2	4.1	0.602
**MLD**	Median	12.7	12.4	12.7	0.463
**PTV volume**	Median	324.5	306.0	318.0	0.414

NSCLC, non-small cell lung cancer; SCLC, small cell lung cancer; MHD, mean heart dose. The bold values represent variables with statistically significant p values within the table.

Ischaemic heart disease was the most common pre-existing cardiac condition, as it was recorded in the HES of 200 (21.9%) of patients. One hundred and fifty-five patients (17.0%) were recorded as having an arrythmia, 28 patients (3.1%) were recorded as having valve disease and 9 patients (1%) with pericardiac or myocardial disease.

### Death with a cardiac cause

There were 110 patients (11.4%) treated with hypofractionated radiotherapy who died with a cardiac cause. The most common cardiac event on death certificates was ischaemic heart disease (7.5%) followed by heart failure (2.6%) and cardiac arrythmia (1.6%) (**Table 3**).

**Table 3:** Number of death certificated mentions of cardiac events.

Cardiac event	Death certificate mentions (% of total patients)
Ischaemic Heart Disease (I20-I25)	73 (7.5%)
Heart failure (I50, I51)	25 (2.6%)
Arrythmia (I44-I49)	15 (1.6%)
Pericardial and myocardial (I30-I32, I40-I43)	6 (0.6%)
Valve (I34-I37)	5 (0.5%)

### Overall survival

Graphs of the scaled Schoenfeld residuals demonstrate that the assumption of proportional hazards is supported for the variables of interest (**Supplementary Figure 1**).

MHD was significantly associated with worse all cause mortality on multivariable cox analysis (adjusted HR 1.03, CI 1.01-1.03, p=0.001) as were heart V5Gy (aHR 1, CI 1.00-1.01, p=0.03), V30Gy (aHR 1.02, CI 1.01-1.03, p=0.001) and V50Gy (aHR 1.03, CI 1.01-1.05, p=0.01). Age at radiotherapy (aHR1.02, CI 1.01-1.03, p=0.001) male sex (aHR 1.21, p=0.02), T4 tumours (aHR 1.50, p=0.004), mean lung dose (aHR 1.04, p=0.002) and deprivation quintile 4 (aHR 1.40, p=0.016) were all associated with increased hazard of death at all heart dose parameters (**Supplementary Table 1**). Presence of a cardiac comorbidity prior to radiotherapy was not associated with all-cause overall survival.

### Death with a cardiac cause

#### Patients with pre-existing cardiac comorbidities

**Figure 2:**
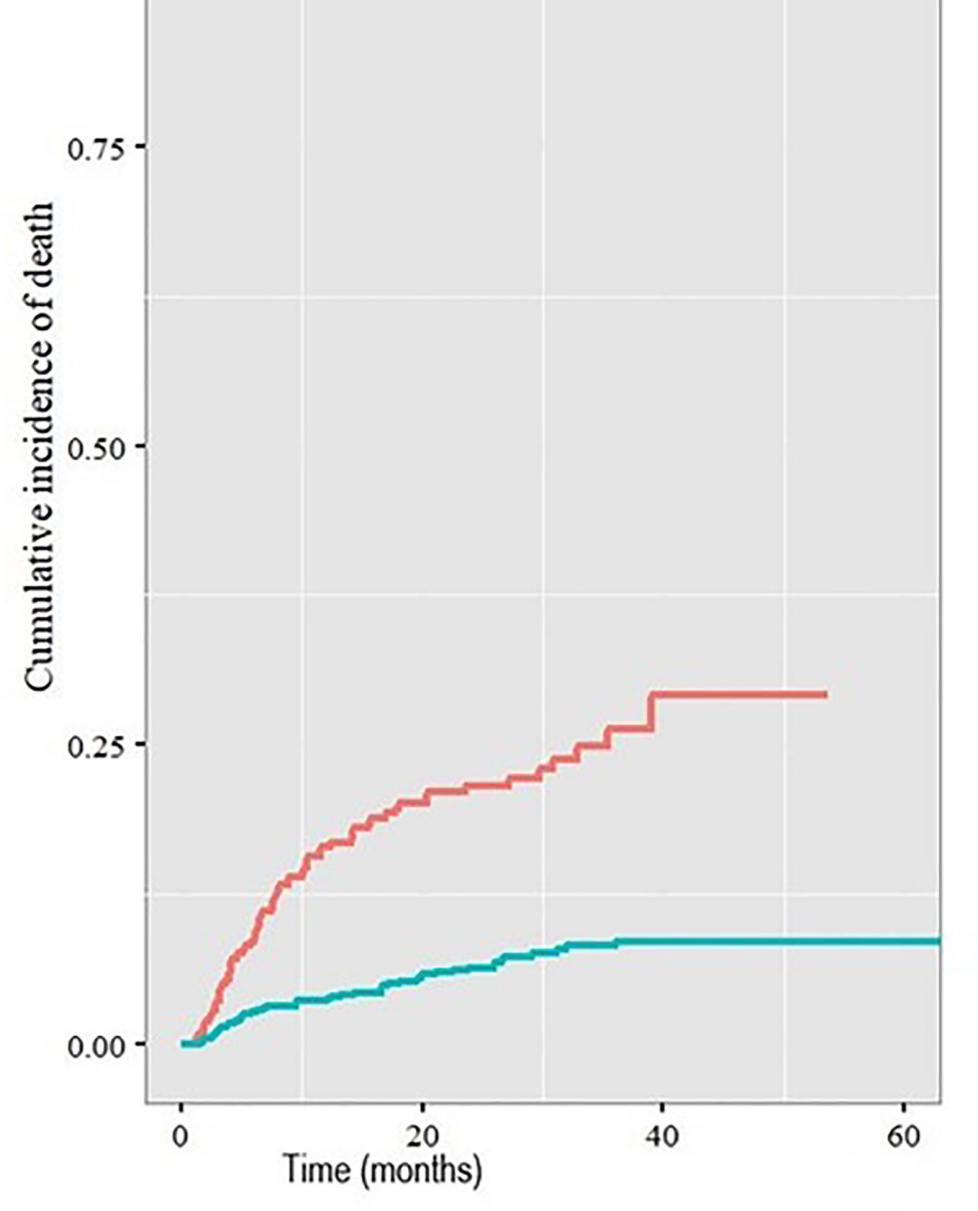
Cumulative incidence of death with a cardiac cause in patients with (red line) and without (blue line) pre-existing cardiac comorbidities.

Patients with a cardiac comorbidity had an increased risk of death with a cardiac cause compared with those without a cardiac comorbidity (2-year cumulative incidence rate 21.3% v 6.2%, p<0.001, **Figure 2**).

**Figure 3:**
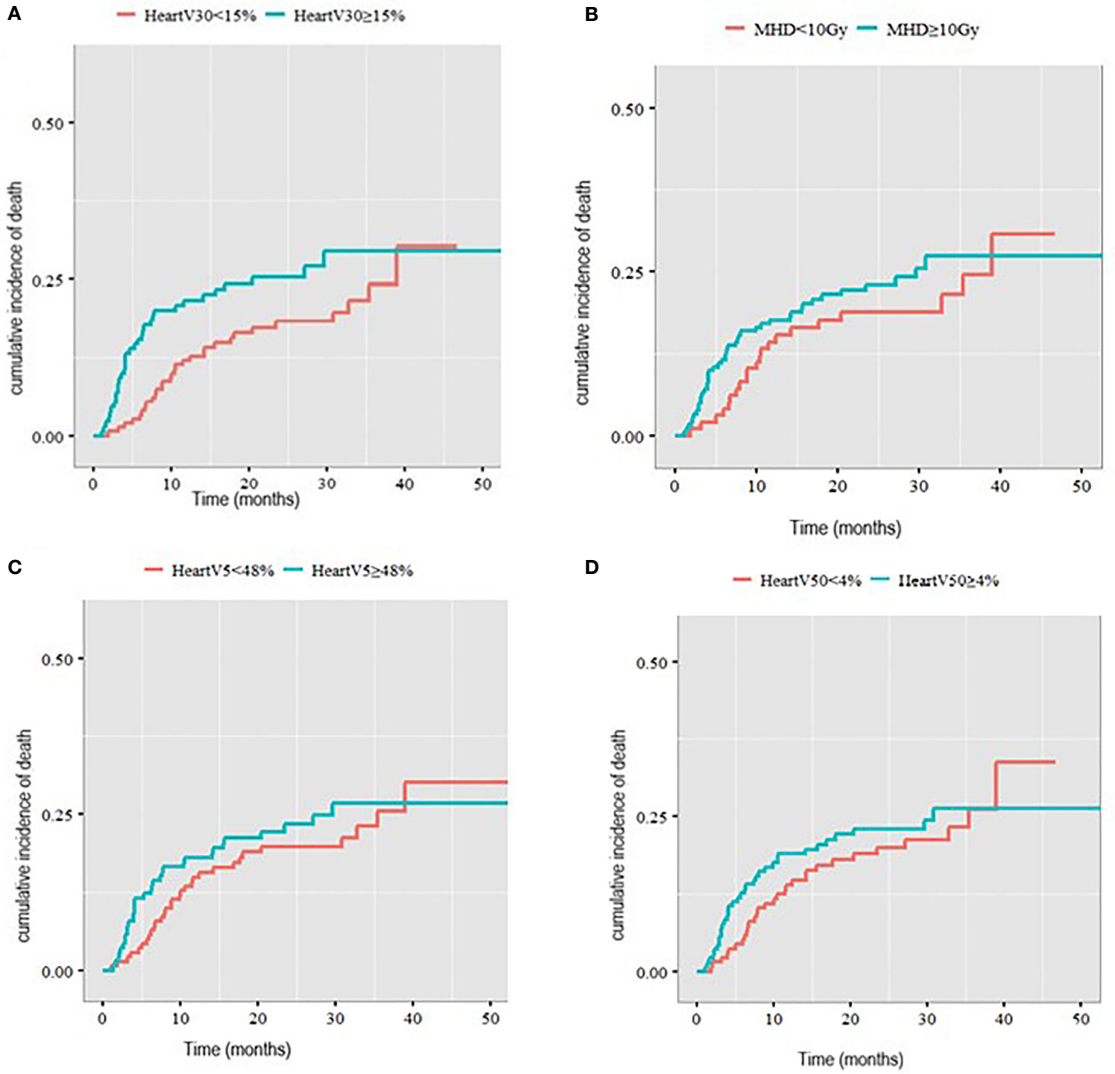
Cumulative incidence of death with a cardiac cause in patients with pre-existing cardiac comorbidities stratified by **(A)** V30Gy, **(B)** mean heart dose, **(C)** heart V5Gy, **(D)** heart V50Gy.

The median heart V30Gy for the whole cohort was 15%. Heart V30Gy ≥ 15% was associated with a significantly higher cumulative incidence of death with a cardiac cause compared to patients with heart V30Gy <15% (2-year rate 25.8% v 17.3%, p=0.05, **Figure 3A**) in patients with a pre-existing cardiac comorbidity.

There was no statistically significant difference in the cumulative incidence of death with a cardiac cause in patients with a cardiac comorbidity when comparing MHD < 10Gy to MHD ≥10Gy (2-year incidence 18.1% v 23.3%, p= 0.3). Nor was there a difference in cumulative incidence of death with a cardiac cause with heart V5Gy split at a median of 48% (2-year cumulative incidence 22.8% compared to 21.4%) and heart V50Gy split at a median of 4% (2-year cumulative incidence 22.6% v 20.1%) (**Figure 3**).

After adjustment for tumour and cardiac risk factors using multivariable Fine and Gray analysis for cardiac death with other causes of death as a competing risk, increasing age at the start of radiotherapy (aHR 2.07, p=0.002), male sex (aHR 1.99, p=0.03) and right sided tumours (aHR 2.71, p=0.004) were associated with an increased risk of death with a cardiac cause for all heart dose parameters (**Table 4**). Higher cardiac V30Gy was associated with an increased risk of death with a cardiac cause (aHR 1.03, 1.00-1.07, p=0.04) but not MHD, cardiac V5Gy or cardiac V50Gy.

**Table 4:** Fine and Gray analysis of death with a cardiac cause in patients with a pre-existing cardiac comorbidity.

Variable	aHR MHD	aHR V5Gy	aHR V30	aHR V50
Age at radiotherapy (continuous)	**1.06 (1.01-1.1, p=0.015)**	**1.05 (1.01-1.1, p=0.021)**	**1.05 (1.01-1.11, p=0.009)**	**1.06 (1.01-1.13, p=0.01)**
Sex (female v male)	**2.01 (1.08-3.75, p=0.02)**	**1.99 (1.06-3.69, p=0.03)**	**2.07 (1.105-2.39, p=0.02)**	**2.07 (1.11-3.56, p=0.02)**
**Performance Status (PS 0 ref)**				
PS 1	1.78 (0.46-6.83, p=0.4)	1.56 (0.43-5.64, p=0.5)	1.55 (0.42-5.74, p=0.52)	1.62(0.44-5.98, p=0.19)
PS 2	1.90 (0.52-6.81, p=0.33)	1.81 (0.54-6.1, p=0.34)	1.97 (0.56-6.94, p=0.29)	2.04 (0.58-7.18, p=0.27)
PS 3	2.96 (0.71-12.38, p=0.14)	2.91 (0.73-11.61, p=0.13)	2.88 (0.69-11.99, p=0.74)	3.4 (0.83-13.95, p=0.09)
**T stage (T1 ref)**				
T2	0.44 (0.19-1.02, p=0.06)	**0.45 (0.20-0.98, p=0.04)**	**0.43 (0.19-0.93, p=0.03)**	**0.43 (0.20-0.94, p=0.03)**
T3	0.5 (0.23-1.1, p=0.08)	0.47 (0.22-1.03, p=0.06)	**0.42 (0.20-0.93, p=0.03)**	**0.44 (0.2-0.95, p=0.04)**
T4	0.61 (0.25-1.5, p=0.28)	0.57 (0.23-1.41, p=0.22)	0.52 (0.22-1.2, p=0.13)	0.52 (0.22-1.22, p=0.13)
**N stage (N0 ref)**				
N1	0.61 (0.24-1.54, p=0.29)	0.67 (0.27-1.67, p=0.38)	0.55 (0.22-1.40, p=0.21)	0.59 (0.23-1.51, p=0.27)
N2	1.41 (0.70-2.81, p=0.75)	1.56 (0.79-3.07, p=0.2)	1.22 (0.61-2.45, p=0.58)	1.29 (0.64-2.59, p=0.48)
N3	0.82 (0.24-2.18, p=0.75)	0.95 (0.26-3.49, p=0.94)	0.74 (0.21-2.63, p=0.64)	0.77 (0.22-2.72, p=0.68)
**Deprivation quintile (Q1, least deprived ref)**				
2	0.74 (0.16-3.39, p=0.69)	0.78 (0.18-3.6, p=0.77)	0.84 (0.19-3.84, p=0.82)	0.85 (0.18-3.98, p=0.84)
3	1.65(0.62-4.39, p=0.31)	1.79 (0.7-4.61, p=0.23)	1.79 (0.21-2.63, p=0.64)	1.79 (0.70-4.56, p=0.22)
4	2.39 (0.97-5.9, p=0.06)	2.39 (0.97-5.92, p=0.06)	**2.53 (1.03-6.25, p=0.04)**	**2.51 (1.02-6.18, p=0.05)**
5	1.38 (0.52-3.71, p=0.52)	1.44 (0.54-3.84, p=0.47)	1.34 (0.50-3.61, p=0.56)	1.40 (0.52-3.79, p=0.51)
Laterality (left v right)	**2.72 (1.37-5.41, p=0.004)**	**2.56 (1.27-5.16, p=0.008)**	**2.71 (1.39-5.28, p=0.004)**	**2.65 (1.37-5.13, p=0.004)**
Chemotherapy prior to radiotherapy (no v yes)	0.85 (0.33-2.16, p=0.73)	0.98 (0.40-2.39, p=0.97)	0.97 (0.40-2.35, p=0.95)	1.00 (0.42-2.40, p=0.99)
Heart dose parameter	1.04 (0.99-1.09, p=0.13)	1.01 (0.99-1.02, p=0.43)	**1.03 (1.00-1.07, p=0.04)**	1.06 (0.99-1.14, p=0.10)
Mean lung dose	0.98 (0.89-1.08, p=0.69)	1.00 (0.91-1.11, p=0.96)	0.94 (0.85-1.04, p=0.21)	1.00 (0.93-1.09, p=0.91)

Number of patients analysed = 273. The bold values represent variables with statistically significant p values within the table.

#### Patients without a pre-existing cardiac comorbidity

There was no significant difference in 2-year cumulative incidence of death with a cardiac cause in patients without a cardiac comorbidity for MHD, cardiac V30Gy, V5Gy or V50Gy. The 2-year cumulative incidence of death with a cardiac cause in patients without a cardiac comorbidity was 5.1% in patients with heart V30Gy <15% and 6.4% in patients with heart V30Gy≥ 15% (p=0.45). For patients with MHD<10Gy 2-year cumulative incidence of death with a cardiac cause was 4.5% compared to 6.9% for patients with MHD ≥10Gy (p=0.29). Cumulative incidence curves are shown in **Supplementary Figure 2**.

After adjusting for tumour and cardiac risk factors, increasing age at the start of radiotherapy and cardiac dosimetric parameters were associated with death with a cardiac cause (**Table 5**). The adjusted HR for death with a cardiac cause for MHD was 1.07 (1.01-1.13, p=0.021), for heart V5Gy 1.01 (1-1.13, p=0.05) and for heart V30Gy 1.04 (1-1.07, p=0.039).

**Table 5:** Fine and Gray analysis of death with a cardiac cause in patients with no cardiac comorbidity.

Variable	aHR MHD	aHR V5Gy	aHR V30Gy	aHR V50Gy
Age at radiotherapy (continuous)	**1.05 (1.02-1.09, p=0.004)**	**1.05(1.01-1.09, p=0.008)**	**1.05 (1.01-1.1, p=0.008)**	**1.05 (1.01-1.08, p=0.01)**
Sex (female v male)	1.21 (0.62-2.35, p=0.57)	1.23 (0.64-2.38, p=0.53)	1.22 (0.62-2.39, p=0.56)	1.22 (0.06-2.35, p=0.55)
**Performance Status (PS 0 ref)**				
PS 1	0.38 (0.09-1.53, p=0.17)	0.72 (0.09-1.50, p=0.16)	0.38 (0.09-1.64, p=0.2)	0.38 (0.09-1.61, p=0.19)
PS 2	0.80 (0.21-3.02, p=0.75)	0.81 (0.21-3.06, p=0.75)	0.80 (0.2-3.14, p=0.74)	0.81 (0.21-3.16, p=0.76)
PS 3	0.78 (0.15-3.95, p=0.76)	0.75 (0.15-3.85, p=0.73)	0.75 (0.14-3.93, p=0.74)	0.71 (0.13-3.7, p=0.76)
**T stage (T1 ref)**				
T2	0.91 (0.32-2.56, p=0.85)	0.52 (0.34-2.60, p=0.90)	0.96 (0.34-2.73, p=0.94)	1.05 (0.37-2.94, p=0.93)
T3	0.95 (0.31-2.95, p=0.93)	1.01 (0.33-3.10, p=0.99)	1.00 (0.33-3.07, p=0.99)	1.088 (0.36-3.2, p=0.88)
T4	1.53 (0.51-4.56, p=0.45)	1.74 (0.57-5.30, p=0.33)	1.54 (0.52-4.58, p=0.44)	1.70 (0.56-5.13, p=0.35)
**N stage (N0 ref)**				
N1	0.89 (0.38-2.07, p=0.78)	0.94 (0.39-2.23, p=0.89)	0.83 (0.35-1.98, p=0.67)	0.92 (0.38-2.21, p=0.85)
N2	0.67 (0.28-1.59, p=0.36)	0.77 (0.32-1.86, p=0.56)	0.58 (0.23-1.48, p=0.56)	0.73 (0.30-1.79, p=0.49)
N3	1.81 (0.64-5.1, p=0.26)	2.06 (0.70-6.02, p=0.19)	1.37 (0.47-3.93, p=0.56)	1.74 (0.61-4.93, p=0.3)
**Deprivation quintile (Q1, least deprived ref)**				
2	0.94 (0.22-4.07, p=0.94)	0.95 (0.22-4.09, p=0.94)	0.97 (0.23-4.17, p=0.97)	0.93 (0.21-3.98, p=0.92)
3	0.64 (0.15-2.62, p=0.53)	0.67 (0.16-2.76, p=0.57)	0.64 (0.15-2.66, p=0.54)	0.65 (0.15-2.70, p=0.55)
4	1.98 (0.63-6.21, p=0.24)	2.09 (0.66-6.67, p=0.21)	1.93 (0.63-5.89, p=0.25)	1.95 (0.62-6.08, p=0.25)
5	1.51 (0.55-2.62, p=0.53)	1.52 (0.55-4.22, p=0.42)	1.60 (0.59-4.35, p=0.36)	1.54 (0.56-4.22, p=0.4)
Laterality (left v right)	1.08 (0.58-1.99, p=0.81)	0.98 (0.53-1.79, p=0.94)	1.08 (0.58-2.00, p=0.81)	0.92 (0.49-1.72, p=0.78)
Chemotherapy prior to radiotherapy (no v yes)	0.21 (0.04-1.04, p=0.06)	0.214 (0.04-1.06, p=0.06)	0.35 (0.12-1.07, p=0.07)	0.20 (0.04-0.99, p=0.05)
Heart dose parameter	**1.07 (1.01-1.13, p=0.021)**	**1.01 (1.00-1.03, p=0.05)**	**1.04 (1.00-1.07, p=0.039)**	1.01 (0.95-1.07, p=0.78)
Mean lung dose	0.92 (0.82-1.03, p=0.13**)**	0.94 (0.84-1.04, p=0.23)	0.94 (0.85-1.04, p=0.21)	0.99 (0.92-1.06, p=0.7)

Number of patients analysed = 631. The bold values represent variables with statistically significant p values within the table.

## Discussion

In line with existing literature this study, combining population data with radiotherapy data, demonstrates how cardiac radiation dose and cardiac comorbidities both contribute to subsequent cardiac mortality in patients undergoing thoracic radiotherapy.

This is the largest published cohort investigating survival, cardiac outcomes and cardiac radiation dose in patients treated with curative hypofractionated radiotherapy for lung cancer, accounting for existing cardiac comorbidities. Unlike other cohorts it includes patients with early-stage disease and the majority of patients (78%) did not receive chemotherapy before or during radiotherapy.

This study adds to existing literature showing that increasing MHD (9, 13, 14, 18), heart V30Gy (19), heart V50Gy (9) and heart V5Gy (12) are all associated with worse overall survival in patients having curative radiotherapy for lung cancer. Moreover, we show that mean lung dose is significantly associated with survival, demonstrating that lung dose remains important in addition to heart dose.

Patients with lung cancer have poor survival due to their disease and comorbidities, therefore this study used data on cause of death from death certificates and Fine and Gray analysis to take account of the competing risk of death from other causes. Moreover, variables of interest were pre-defined prior to analysis based on previous studies to include variables that are important for outcome in lung cancer and cardiac disease to avoid over-fitting. Pre-defined variables allowed consistent analysis across different populations and dose parameters, compared to the technique of variable selection (20) which has been used in other studies (9, 13, 14).

We found that patients with known cardiac comorbidities had a 2-year cumulative incidence of cardiac-related death of 21.3% compared to 6.2% in those without a cardiac comorbidity. Similarly, in their study of 125 patients with lung cancer treated in radiotherapy dose escalation studies, Dess et al. (13) found the rate of grade ≥3 cardiac events at 2 years was 21% in patients with pre-existing cardiac disease and 7% in patients without pre-existing cardiac disease. Atkins et al. (14) describe a lower rate of major adverse cardiac events at 2 years of 11.7% in patients with heart disease and 2.5% in those without heart disease, in a cohort of 748 patients with locally advanced lung cancer treated with radiotherapy.

Both Dess and Atkins found that MHD ≥10Gy was associated with an increased rate of MACE/grade ≥3 cardiac events on univariable analysis in patients with pre-existing cardiac disease. Our study found that although patients with MHD≥10Gy had an increased incidence of cardiac death, at 23.3% compared with 18.1% if MHD<10Gy, this was not statistically significant. Heart V30Gy ≥15% in patients with cardiac comorbidities was the dose parameter significantly associated with an increased incidence of cardiac death in our study, with a 2-year rate of 25.8% compared with 17.3% if heart V30Gy <15%. Once adjustment was made for other cardiac and tumour factors, the association between heart V30Gy and cardiac death remained with an adjusted HR of 1.03 (1-1.07). Increasing age and male sex were associated with cardiac death, and patient deprivation also demonstrated significance in the heart V30Gy model. Male sex, age and deprivation are all know cardiac risk factors (21) demonstrating their contribution to cardiac-related deaths following radiotherapy. The weak association between cardiac dose and death involving a cardiac cause in patients with known cardiac comorbidities may indicate that radiation exposure to the whole heart results in only a small relative increase in a cohort of patients already at high risk of cardiac events. The absolute increase in cumulative incidence of death involving a cardiac cause at 2-years comparing MHD<10Gy vs MHD>10Gy is 5% in patients with a known cardiac comorbidity and 3% in those without a cardiac comorbidity.

In patients without a known cardiac comorbidity, the 2-year cumulative incidence of cardiac death was slightly higher in patient with heart V30Gy ≥ 15% compared to V30Gy < 15% (6.4% v 5.1%) and MHD ≥10Gy compared to MHD < 10Gy (6.9% v 4.6%) however, these were not statistically significant. On adjustment for tumour and patient factors, MHD (HR 1.07, 10.1-1.13), heart V30Gy (HR 1.04, 1.0-1.07) and heart V5Gy (HR 1.01, 1.0-1.03) were associated with death with a cardiac cause. Age was also associated with cardiac-related death in those without cardiac comorbidity however, in contrast to the group with a cardiac comorbidity, tumour laterality, deprivation and male sex were not.

The difference In variables that influence cardiac-related death between the cardiac comorbidity and no cardiac comorbidity groups would seem to suggest that heart dose is of particular importance in patients without a cardiac comorbidity. It is possible, however, that many of these patients have undiagnosed cardiac disease prior to starting treatment which could subsequently manifests after radiotherapy. In addition, patients with a previous cardiac event will receive cardiac risk factor management, as demonstrated by the significantly higher number of ex-smokers in the cardiac comorbidity group. Smoking is known to result in worse survival after radiotherapy (22). Furthermore, cardiac medications such as statins (23) and angiotensin converting enzyme inhibitors, which are prescribed routinely in patients after a cardiac event, may provide protection again radiation induced cardiac toxicity (24).

In patients with a cardiac comorbidity, right sided tumours were associated with cardiac death at all dose parameters tested, with an adjusted HR of 2.56-2.72 depending on which parameter was included in the analysis. Our group has previously shown that patients with right-sided lung tumours have worse overall survival (25) and other studies have found that dose to the right side of the heart is associated with cardiac events and death (26–28). The sino-atrial node, which generates the electrical impulse that stimulates the heart to beat, is located in the superior right atrium and the atrio-ventricular node, which co-ordinates ventricular contraction, is located in the posterior-inferior right atrium. We hypothesise that patients with an already vulnerable heart are particularly sensitive to irradiation of these essential cardiac conduction substructures.

This study only used patients who had a cardiac contour at the time of radiotherapy in order to standardise planning techniques and minimise any impact of improvements in plan optimisation when the heart was included. To overcome this issue, future work should use automatic segmentation of whole heart and substructures to examine impact of substructure dose on outcome as there is conflicting evidence and poor physiological understanding of the radiation sensitive regions of the heart.

The main limitation of this study relates to the shortcomings of using population-based data to identify cardiac comorbidities and outcomes. In our study, patients were only recorded as having a cardiac problem if they had been admitted to hospital and had a relevant entry on Hospital Episode Statistics prior to radiotherapy. Consequently, some patients with cardiac comorbidities may have been missed, although our method will have identified those patients with the most severe cardiac disease. Nevertheless, thirty percent of patients in this cohort were identified using HES as having a cardiac comorbidity which is consistent with existing literature (3, 4, 13, 14)

The 2-year cumulative incidence of death with a cardiac cause in this study is higher than the rates of cardiac events reported in other studies. This is likely due to the different endpoint definitions used in the different studies, as well as differences in study population. We used the endpoint death with a cardiac cause, defined as any mention of a cardiac cause of death on the death certificate. This is the same method that has been used to classify deaths from sepsis (29) and by Public Health England to count deaths related to severe acute respiratory syndrome coronavirus 2 (30), however, it may overestimate the number of cardiac deaths. The alternative method of using only the primary cause of death in analysis will underestimate the contribution of cardiac events in patients with cancer (31–35) as a patient’s death may have multiple contributing causes that are not adequately recorded. A consensus on cardiac event recording following cancer treatment would help to guide future work in cardio-oncology.

In this large, retrospective study of patients treated with radical, hypofractionated radiotherapy for lung cancer we show the complex interplay between patient comorbidities and heart dose in predicting future cardiac events and survival. Pre-existing heart disease is a pre-disposing factor for future cardiac death following radiotherapy, regardless of cardiac dose, therefore, cardiac surveillance should be considered before and after treatment. Further work incorporating cardiac medication data is required to understand the potential protective effects of medications in patients having thoracic radiotherapy. Secondly, stricter heart dose parameters should be used in thoracic radiotherapy. The quantitative analysis of normal tissue effects in the clinic (QUANTEC) suggests V30Gy <46% however, our study shows that V30Gy <15% may be a more appropriate threshold, especially in patients with pre-existing cardiac disease. Achieving stricter heart dose parameters may be possible with advanced technologies such as proton therapy. Further understanding of the radiation sensitivity of cardiac substructures, such as the base of the heart containing the conduction system, in patients with and without cardiac risk factors will enable better cardiac sparing in the future.

## Data availability statement

KB, CF-F and AMcW conceived the study. FS, AMcW and KF obtained data and ethical approvals from Public Health England. KB and AA analysed the data. KB wrote the manuscript with assistance from all authors. All authors contributed to the article and approved the submitted version.

## Ethics statement

The studies involving human participants were reviewed and approved by Leeds East Research Ethics Committee (18/YH/0058). The ethics committee waived the requirement of written informed consent for participation.

## Author contributions

KB, CF-F and AMcW conceived the study. FS, AMcW and KF obtained data and ethical approvals from Public Health England. KB and AA analysed the data. KB wrote the manuscript with assistance from all authors. All authors contributed to the article and approved the submitted version.

## Funding

This work is supported by Yorkshire Cancer Research [M401] and by Cancer Research UK via funding to the Cancer Research UK Manchester Centre [C147/A25254] and their Clinical Academic Training Award [C19941/A28707]. CF-F and MvH are supported by NIHR Manchester Biomaedical Research Centre.

## Conflict of interest

The authors declare that the research was conducted in the absence of any commercial or financial relationships that could be construed as a potential conflict of interest.

## Publisher’s note

All claims expressed in this article are solely those of the authors and do not necessarily represent those of their affiliated organizations, or those of the publisher, the editors and the reviewers. Any product that may be evaluated in this article, or claim that may be made by its manufacturer, is not guaranteed or endorsed by the publisher.
