# Supplementary material for: Heart dose and cardiac comorbidities influence death with a cardiac cause following hypofractionated radiotherapy for lung cancer
Source: Front Oncol. 2022 Oct 11;12:1007577. doi: 10.3389/fonc.2022.1007577 (PMC9592751; doi:10.3389/fonc.2022.1007577)
Supplement: Supplementary file 1 [file DataSheet_1.docx]

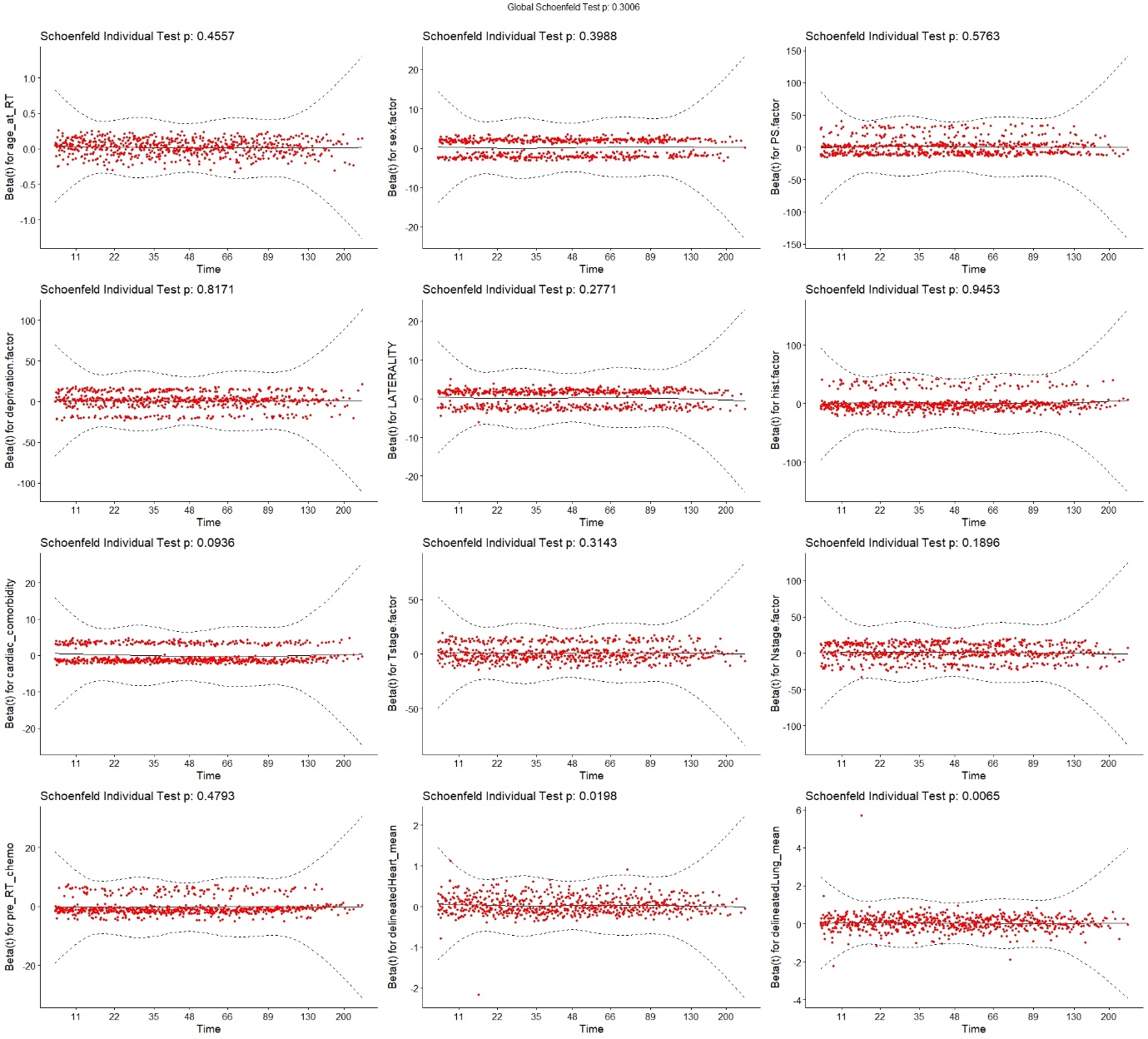


Supplementary figure 1 graphical representation of Schoenfeld residuals for variables in Cox model

| **Variable** | **aHR MHD** | **aHR V30Gy** | **aHR V50Gy** | **aHR V5Gy** |
| --- | --- | --- | --- | --- |
| Age (continuous) | **1.02 (1.01-1.03, p<0.001)** | **1.02 (1.01-1.03, p<0.001)** | **1.02 (1.01-1.03, p=0.001)** | **1.02 (1.01-1.02, p=0.001)** |
| Sex (female v male) | **1.21 (1.03-1.42, p=0.022)** | **1.20 (1.03-1.41, p=0.023)** | **1.21 (1.03-1.42, p=0.022)** | **1.21 (1.03-1.42, p=0.020)** |
| **Performance Status (PS 0 ref)** | | | | |
| PS 1 | 0.84 (0.62-1.13, p=0.241) | 0.85 (0.63-1.14, p=0.275) | 0.83 (0.62-1.12, p=0.217) | 0.83 (0.62-1.13, p=0.235) |
| PS 2 | 0.91 (0.66-1.24, p=0.544) | 0.93 (0.68-1.27, p=0.649) | 0.91 (0.67-1.24, p=0.560) | 0.90 (0.66-1.23, p=0.516) |
| PS 3 | 1.09 (0.74-1.62, p=0.665) | 1.12 (0.75-1.66, p=0.577) | 1.07 (0.73-1.59, p=0.720) | 1.07 (0.72-1.58, p=0.749) |
| **Histology (NSCLC ref)** | | | | |
| No histology (clinical diagnosis) | 0.98 (0.76-1.27, p=0.898) | 0.98 (0.75-1.26, p=0.850) | 0.95 (0.74-1.23, p=0.716) | 0.97 (0.75-1.25, p=0.804) |
| SCLC | 1.24 (0.98-1.58, p=0.077) | 1.20 (0.94-1.53, p=0.137) | 1.25 (0.98-1.59, p=0.069) | 1.27 (1.00-1.62, p=0.050) |
| **T stage (T1 ref)** | | | | |
| T2 | 1.13 (0.87-1.46, p=0.374) | 1.15 (0.89-1.49, p=0.295) | 1.16 (0.89-1.50, p=0.263) | 1.14 (0.88-1.48, p=0.324) |
| T3 | 1.23 (0.94-1.62, p=0.133) | 1.25 (0.95-1.64, p=0.109) | 1.29 (0.99-1.69, p=0.063) | 1.27 (0.96-1.66, p=0.091) |
| T4 | **1.52 (1.15-2.00, p=0.003)** | **1.50 (1.14-1.98, p=0.004)** | **1.51 (1.15-2.00, p=0.003)** | **1.57 (1.19-2.07, p=0.001)** |
| **N stage (N0 ref)** | | | | |
| N1 | 0.90 (0.71-1.14, p=0.378) | 0.86 (0.68-1.10, p=0.232) | 0.90 (0.71-1.15, p=0.404) | 0.94 (0.74-1.19, p=0.608) |
| N2 | 1.16 (0.94-1.43, p=0.163) | 1.08 (0.86-1.34, p=0.515) | 1.15 (0.93-1.42, p=0.209) | 1.25 (1.01-1.53, p=0.039) |
| N3 | 1.08 (0.81-1.44, p=0.615) | 0.97 (0.72-1.29, p=0.818) | 1.02 (0.77-1.36, p=0.875) | 1.12 (0.84-1.50, p=0.452) |
| **Deprivation quintile (Q1, least deprived ref)** | | | | |
| 2 | 1.28 (0.92-1.79, p=0.147) | 1.28 (0.92-1.79, p=0.145) | 1.28 (0.91-1.78, p=0.152) | 1.27 (0.91-1.77, p=0.164) |
| 3 | 1.15 (0.86-1.54, p=0.347) | 1.15 (0.86-1.54, p=0.348) | 1.17 (0.87-1.57, p=0.289) | 1.15 (0.86-1.54, p=0.346) |
| 4 | **1.40 (1.06-1.83, p=0.016)** | **1.38 (1.05-1.81, p=0.020)** | **1.34 (1.02-1.75, p=0.036)** | **1.37 (1.05-1.80, p=0.022)** |
| 5 | 1.23 (0.96-1.58, p=0.104) | 1.23 (0.95-1.58, p=0.113) | 1.21 (0.95-1.56, p=0.128) | 1.23 (0.95-1.58, p=0.113) |
| Laterality (left v right) | 1.15 (0.98-1.35, p=0.093) | 1.18 (1.00-1.39, p=0.051) | 1.13 (0.96-1.33, p=0.135)- | 1.10 (0.94-1.29, p=0.241) |
| Pre-radiotherapy cardiac comorbidity (no v yes) | 1.00 (0.85-1.19, p=0.968) | 1.01 (0.85-1.20, p=0.905) | 1.01 (0.85-1.19, p=0.938) | 1.00 (0.85-1.19, p=0.961) |
| Chemotherapy prior to radiotherapy (no v yes) | 0.81 (0.65-1.01, p=0.057) | 0.81 (0.66-1.01, p=0.060) | 0.79 (0.64-0.98, p=0.030) | 0.80 (0.64-0.99, p=0.040) |
| Heart dose parameter | **1.03 (1.01-1.05, p<0.001)** | **1.02 (1.01-1.03, p<0.001)** | **1.03 (1.01-1.05, p=0.010)** | **1.00 (1.00-1.01, p=0.026)** |
| Mean lung dose | **1.04 (1.01-1.07, p=0.015)** | **1.04 (1.02-1.07, p=0.002)** | **1.06 (1.03-1.08, p<0.001)** | **1.05 (1.03-1.08, p<0.001)** |

Supplementary table 1 Cox regression analysis for overall survival and cardiac dose parameters for all patients (Number of patients analysed = 941)


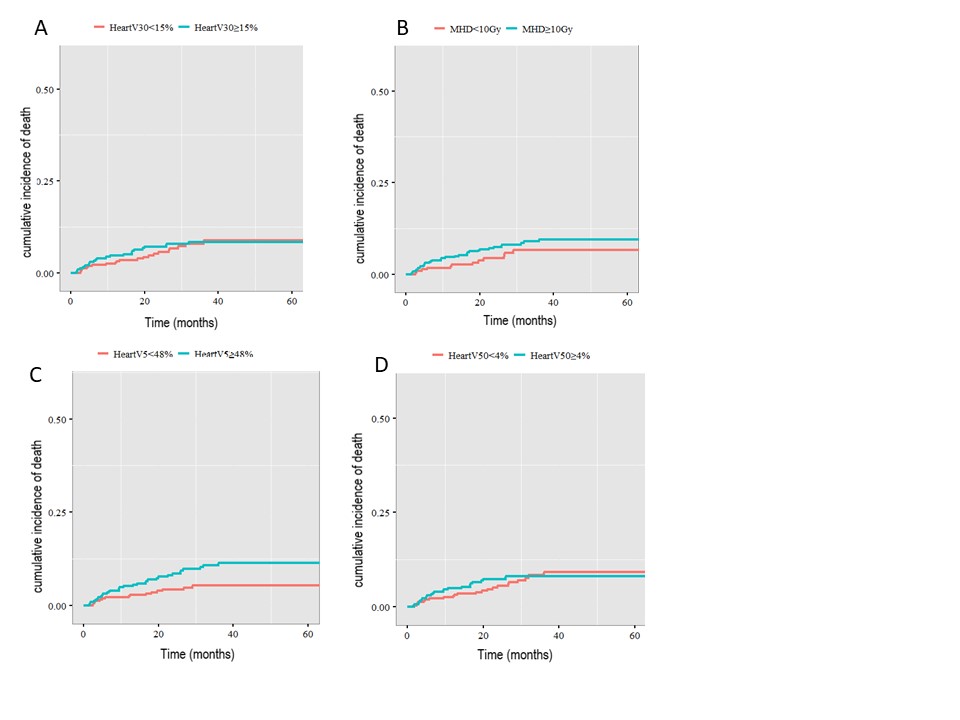


Supplementary Figure 2 Cumulative incidence of death with a cardiac cause in patients with no cardiac comorbidities stratified by (A) MHD, (B) heart V30Gy, (C) heart V5Gy, (D) heart V50Gy
